# Clinical outcomes of fetuses with chromosome 16 short arm copy number variants

**DOI:** 10.1002/mgg3.2174

**Published:** 2023-04-04

**Authors:** Jessica Kang, Chien‐Nan Lee, Yi‐Ning Su, Yi‐Yun Tai, Chih‐Ling Chen, Han‐Ying Chen, Shin‐Yu Lin

**Affiliations:** ^1^ Department of Obstetrics and Gynecology National Taiwan University Hospital Taipei Taiwan; ^2^ Dianthus Maternal Fetal Medicine Clinic Sofiva Genomics Co., Ltd. Taipei Taiwan; ^3^ Department of Medical Genetics National Taiwan University Hospital Taipei Taiwan

**Keywords:** chromosome 16, copy number variants, genetic counseling, prenatal diagnosis

## Abstract

**Background:**

The short arm of chromosome 16 consists of several copy number variants (CNVs) that are crucial in neurodevelopmental disorders; however, incomplete penetrance and diverse phenotypes after birth aggravate the difficulty of prenatal genetic counseling.

**Methods:**

We screened 15,051 pregnant women who underwent prenatal chromosomal microarray analysis between July 2012 and December 2017. Patients with positive array results were divided into four subgroups based on the type of mutation identified on screening (16p13.3, 16p13.11, 16p12.2, and 16p11.2), and the maternal characteristics, prenatal examinations, and postnatal outcomes of different cases were reviewed.

**Results:**

Chromosome 16 CNVs were identified in 34 fetuses, including four with 16p13.3 CNVs, 22 with 16p13.11 CNVs, two with 16p12.2 microdeletions, and six with 16p11.2 CNVs. Of the 34 fetuses, 17 delivered without early childhood neurodevelopmental disorders, three developed neurodevelopmental disorders during childhood, and 10 were terminated.

**Conclusion:**

Incomplete penetrance and variable expressivity make prenatal counseling challenging. Most cases with inherited 16p13.11 microduplication were reported to have normal development in early childhood, and we also report a few cases of de novo 16p CNVs without further neurodevelopmental disorders.

## Graphical abstract

Incomplete penetrance and variable expressivity make prenatal counseling challenging for chromosome 16 copy number variants (CNVs). Most cases with inherited 16p13.11 microduplication were reported to have normal development in early childhood, and we also report a few cases of de novo 16p CNVs without further neurodevelopmental disorders.

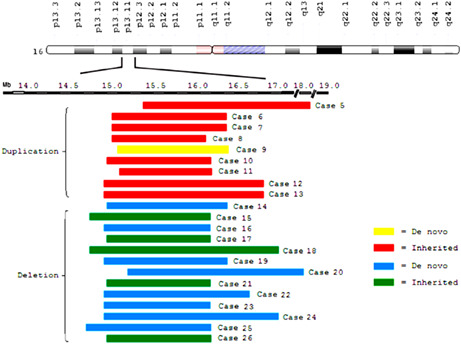

## 1 INTRODUCTION

Genetic approaches are beneficial for understanding neurodevelopmental disorders. Chromosome 16 is gene‐rich and is relatively unstable, causing misalignment of low‐copy repeats during non‐allelic homologous recombination between neighboring intrachromosomal segmental duplications, which accounts for 10% of this chromosome (Redaelli et al., 2019). Previous studies have revealed that copy number variants (CNVs) at the 16p11.2, 16p12.2, and 16p13.11 loci are commonly involved in and associated with diverse phenotypes (Allach El Khattabi et al., 2020; Steinman et al., 2016).

Increased use of chromosome microarray analysis in prenatal genetic testing has caused an incidental increase in the population diagnosed with chromosome 16 CNVs. Although the phenotypic features associated with some of these CNVs have been reported in postnatal or affected cases, it is difficult to perform prenatal counseling due to the rarity of the case and the difficulty in identifying the phenotype prenatally. Incomplete penetrance and variable expressivity are also challenging.

### 1.1 Microdeletion/microduplication of 16p13.3

Rubinstein‐Taybi syndrome (RTS, OMIM #180849) is also called 16p13.3 microdeletion syndrome and involves a microdeletion in the gene encoding the cAMP response element‐binding protein (*CREBBP*) (Breuning et al., 1993). Duplication involving this RTS region is an emerging syndrome called 16p13.3 microduplication syndrome. Its clinical features include congenital anomalies, typical facial and skeletal characteristics, and moderate developmental delay (DD) with intellectual disability (ID) (Demeer et al., 2013; Li et al., 2013; Mattina et al., 2012).

Alpha‐thalassemia is another autosomal recessive disorder involving chromosome 16 (Deisseroth et al., 1977), while α‐thalassemia/mental retardation syndrome of chromosome 16 (ATR16) is associated with very large deletions in 16p13.3 (ATR16 syndrome, OMIM:141750) (Gibson et al., 2008; Wilkie et al., 1990), including deletion in the hemoglobin subunit alpha 1 (*HBA1*; OMIM 141800) and hemoglobin subunit alpha 2 (*HBA2*; OMIM 141850) genes. The clinical features of ATR16 syndrome include ID, hypertelorism, broad or prominent nasal bridge, and talipes equinovarus (Breuning et al., 1993).

### 1.2 Microdeletion/microduplication of 16p13.11

The size of the common 16p13.11 microdeletion varies, but typically involves a 14.7 Mb to 16.3 Mb region. The 16p13.11 region includes several genes, such as mitochondrial inner membrane protein like (*MPV17L*), chromosome 16 open reading frame 45, nuclear distribution gene E homolog 1 (*NDE1*), myosin heavy chain 11 (*MYH11*), and ATP‐binding cassette subfamily C member 1 (*ABCC1*). *NDE1* encodes for nude neurodevelopment protein 1, which is essential for mitosis and neurodevelopment in patients with schizophrenia and other major psychiatric disorders (Bradshaw et al., 2009). NDE1 deficiency impairs neurogenesis, resulting in extreme microcephaly and lissencephaly (Alkuraya et al., 2011; Bakircioglu et al., 2011; Paciorkowski et al., 2013).

The 1.5 Mb deletions of 16p13.11 have been associated with a variety of phenotypes, such as autism, mental retardation, schizophrenia, attention‐deficit hyperactivity disorder (ADHD), ID, microcephaly, and epilepsy. It is the most common genetic risk factor related to seizures and has also been identified in normal individuals. Severe microcephaly, agenesis of the corpus callosum, scalp rugae, and fetal brain disruption‐like phenotypes have been reported with inherited deletions of 16p13.11 (Paciorkowski et al., 2013).

The clinical significance of 16p13.11 duplications is still controversial, despite their frequent detection in patients with cognitive impairment such as DD, schizophrenia, and behavioral impairment such as ADHD. Skeletal and cardiac malformations have also been reported (Nagamani et al., 2011), and a recent report showed a significant risk of cardiovascular disease (Allach El Khattabi et al., 2020). Furthermore, clinical features, such as speech delay and learning disabilities have also been reported in patients with 16p13.11 duplications, and a high percentage of up to 90% of patients inherit the mutation from a parent. A previous study also found it to be a risk factor for thoracic aortic aneurysms and dissection (Kuang et al., 2011).

### 1.3 Microdeletion of 16p12.2

The 16p12.2 microdeletion is approximately a 520 kb deletion with variable expressivity and incomplete penetrance (Rosenfeld et al., 2013) and has been proven to be pathogenic and associated with ID and DD. It has been associated with neurodevelopmental disorders, including cognitive impairment and behavioral problems (Girirajan et al., 2010), as well as, cardiac problems, hearing loss, and renal and cleft lip (Khau Van Kien et al., 2005). A recent case report described an unusual finding of tetralogy of Fallot in a 16p12.2 deletion patient (Linnane et al., 2021).

### 1.4 Microdeletion/microduplication of 16p11.2

Chromosome 16p11.2 deletions and duplications are discovered in approximately 1% of the autism spectrum disorder (ASD) population and are considered the most frequent genetic etiologies (Steinman et al., 2016; Weiss et al., 2008). It is also common in patients with other neurodevelopmental diagnoses, including DDs, ID, and congenital anomalies. The 16p11.2 recurrent microdeletion has a prevalence of approximately 0.03%, and its penetrance in a large cohort subjected to postnatal chromosome microarray analysis was estimated to be 46.8% (Rosenfeld et al., 2010).

Chromosome 16 deletions and duplications share partially mirroring phenotypes. Deletions are associated with ASD, ID, seizures/epilepsy, diabetes‐independent obesity, congenital malformation including spine, brain, and cardiovascular system, such as macrocephaly, while duplications are associated with autism, schizophrenia, anorexia, and microcephaly (Bernier et al., 2017; Steinman et al., 2016).

In this study, we reviewed the prenatal array comparative genomic hybridization (aCGH) results of 15,051 pregnant women. We retrospectively reviewed 34 cases that were diagnosed with CNVs of chromosome 16 short arm and data on their prenatal examinations, postnatal newborn characteristics, and follow‐up clinical condition in childhood. This study aimed to establish the association between intrauterine phenotype and these CNVs and improve the quality of prenatal genetic counseling.

## 2 MATERIALS AND METHODS

### 2.1 Ethical compliance

All the research methods used in this study were approved by the National Taiwan University Hospital Research Ethics Committee (201801010RINC).

### 2.2 Patient characteristics

Prenatal microarray analyses of 15,051 cases were performed at Sofiva Genetics and National Taiwan University Hospital from July 1, 2012, to December 31, 2017. We collected data from 34 pregnant women whose microarray analyses showed that the fetuses carried CNVs involving the short arm of chromosome 16. Clinical information and pregnancy outcomes were collected from the medical records, including maternal characteristics, family history, indications for invasive testing, prenatal ultrasound findings, delivery mode, newborn characteristics, and developmental follow‐up. Indications for invasive testing include advanced maternal age, karyotype abnormalities, abnormal ultrasound findings, and maternal anxiety. Microarray data of all patients were analyzed retrospectively for microdeletions and microduplications involving the short arm of chromosome 16 (16p13.3, 16p13.11, 16p12.2, 16p11.2). The initial developmental status of the patients was assessed by pediatricians during regular follow‐up for vaccination.

The patients underwent amniocentesis or chorionic villus sampling, where 10 mL of amniotic fluid or chorionic villi were sampled through abdominal puncture under ultrasound guidance. Genomic DNA was extracted from the amniotic fluid or chorionic villi using a DNA Extraction Kit (QIAamp® DNA Blood Mini Kit) according to the manufacturer's instructions.

#### 2.2.1 Cytogenomic microarray analysis

An 8 × 60 K oligonucleotide array (Agilent Technologies) was used, and all procedures were performed according to the manufacturer's protocols.

A SurePrint G3 Human CGH Microarray Kit 8 × 60 K (Agilent Technologies) was used. DNA extraction was performed using a QIAamp DNA Blood Mini Kit (QIAGEN). Slides were scanned using the SureScan Microarray Scanner (Agilent Technology) and analyzed with Feature Extraction Software v11.5 (Agilent Technology) under the designed parameters of the human reference genome hg19. Data analysis was conducted using Agilent Cytogenomics software available on the company's website (https://www.genomics.agilent.com/en/CGH‐Microarray‐Data‐Analysis/CytoGenomics‐Software/?cid=AG‐PT‐111&tabId=AG‐PR‐1017, Agilent Cytogenomics v2.7.8.0).

## 3 RESULTS

In total, 15,051 pregnant women prenatally received aCGH, and chromosome 16 CNVs were identified in 34 fetuses. Four cases were diagnosed as 16p13.3 CNVs, 22 as 16p13.11 CNVs, two as 16p12.2 microdeletions, and six as 16p11.2 CNVs, representing 0.03% (4/15051), 0.14% (22/15051), 0.02% (2/15051), and 0.04 (6/15051) of the cases analyzed, respectively.

Of the 34 cases identified to have CNVs, 17 were delivered without further neurodevelopmental outcomes, three were delivered with neurodevelopmental disorders in their childhood, 10 received termination of pregnancy, and four were lost to follow‐up (Table 1).

**TABLE 1:** Findings of fetuses with chromosome 16 CNV and newborn characteristics.

Case	Sex	Dup/del	Size (Mb)	Origin	Prenatal ultrasound finding	Growth IUGR	Delivery mode	Postnatal finding	Follow‐up years	DD
16p13.3										
1	M	Dup	1.03	De novo	−	−	TOP	−	N/A	N/A
2	F	Del	0.01	Pat/Mat	Cardiomegaly	−	TOP	−	N/A	N/A
3	M	Del	0.01	Pat/Mat	−	−	TOP	−	N/A	N/A
4	F	Del	0.54	De novo	−	−	TOP	−	N/A	N/A
16p13.11										
5	M	Dup	3.27	Pat	−	−	VD	Neonatal jaundice	3	−
6	M	Dup	1.26	Mat	−	−	VD	−	4	−
7	F	Dup	1.26	Pat	−	−	VD	−	4	−
8	F	Dup	1.08	Pat	−	−	VD	−	3	−
9	M	Dup	1.16	De novo	−	−	N/A	N/A	N/A	N/A
10	M	Dup	1.23	Pat	−	−	VD	−	4	−
11	M	Dup	1.05	Pat	−	−	N/A	N/A	N/A	N/A
12	M	Dup	1.83	Pat	Cleft lip	−	TOP	−	N/A	N/A
13	M	Dup	1.83	Mat	−	−	VD	−	3	−
14	F	Del	1.34	De novo	−	−	TOP	−	N/A	N/A
15	F	Del	1.43	Mat	EIF	−	N/A	N/A	N/A	N/A
16	M	Del	1.28	De novo	−	−	C/S	−	3	−
17	F	Del	1.23	Mat	−	−	C/S	Café‐au‐lait spot	4	+
18	M	Del	2.1	Mat	−	−	N/A	N/A	N/A	N/A
19	F	Del	1.4	De novo	−	−	TOP	−	N/A	N/A
20	M	Del	3.35	De novo	−	−	C/S	−	4	−
21	F	Del	1.23	Mat	−	−	VD	−	4	−
22	F	Del	1.68	De novo	−	−	TOP	−	N/A	N/A
23	F	Del	1.28	De novo	−	−	C/S	−	3	−
24	F	Del	1.95	De novo	−	−	VD	−	4	+
25	M	Del	1.6	De novo	−	−	TOP	−	N/A	N/A
26	M	Del	1.34	Mat	−	−	C/S	−	4	−
16p12.2										
27	F	Del	1.33	De novo	−	−	VD	−	5	−
28	F	Del	0.57	Mat	Oligohydramnios	+	VD	−	5	−
16p11.2										
29	F	Dup	2.24	De novo	−	+	VD	SGA	4	−
30	M	Dup	0.52	De novo	−	−	VD	−	3	−
31	M	Del	0.52	De novo	−	−	C/S	−	4	−
32	M	Del	0.52	De novo	−	−	TOP	−	4	−
33	M	Del	0.52	De novo	−	−	C/S	−	4	+
34	M	Del	0.52	De novo	−	−	VD	−	3	−

Abbreviations: C/S, cesarean section; DD, developmental delay; Del, deletion; Dup, duplication; EIF, echogenic intracardiac focus; IUGR, intrauterine growth restriction; N/A, not applicable; Pat, paternal; Mat: maternal; SGA, small of gestational age; TOP, termination of pregnancy; VBAC, vaginal birth after cesarean; VD, vaginal delivery.

Reviewing the origin of CNVs, 15 were determined to be inherited from phenotypically normal parents, one of which developed a language delay in her childhood. The other 18 fetuses had de novo mutations, and two of the babies were diagnosed with DD. The residual case was of an unknown origin (Table 2).

**TABLE 2:** Cytogenetic results of chromosome 16.

Case	Dup/del	Region involved	Size (bp)	Boundaries	Karyotype	Origin	Phenotype of parent with CNV
1	Dup	16p13.3	1,027,478	chr16:2,636,140‐3,663,618	N/A	De novo	N/A
2	Del	16p13.3	14,232	chr16:215,724‐229,956	46,XX	N/A	N/A
3	Del	16p13.3	16,961	chr16:215,724‐232,685	46,XX	Paternal/maternal	Normal
4	Del	16p13.3	541,727	chr16:2,312,986‐2,854,713	N/A	De novo	N/A
5	Dup	16p13.11	3,265,214	chr16:15,404,482‐18,669,696	N/A	Paternal	Normal
6	Dup	16p13.11	1,262,260	chr16:15,048,781‐16,311,041	N/A	Maternal	Normal
7	Dup	16p13.11	1,262,260	chr16:15,048,781‐16,311,041	46,XX	Paternal	N/A
8	Dup	16p13.11	1,079,067	chr16:15,048,781‐16,127,848	46,XX	Paternal	Normal
9	Dup	16p13.11	1,162,055	chr16:15,148,986‐16,311,041	N/A	De novo	N/A
10	Dup	16p13.11	1,225,723	chr16:14,968,855‐16,194,578	N/A	Paternal	Normal
11	Dup	16p13.11	1,045,622	chr16:15,148,956‐16,194,578	N/A	Paternal	Normal
12	Dup	16p13.11	1,831,955	chr16:14,910,228‐16,742,183	N/A	Paternal	Normal
13	Dup	16p13.11	1,831,955	chr16:14,910,228‐16,742,183	N/A	Maternal	Normal
14	Del	16p13.11	1,342,163	chr16:14,968,878‐16,311,041	N/A	De novo	N/A
15	Del	16p13.11	1,432,280	chr16:14,762,269‐16,194,549	N/A	Maternal	Normal
16	Del	16p13.11	1,284,321	chr16:14,910,228‐16,194,549	N/A	De novo	N/A
17	Del	16p13.11	1,225,671	chr16:14,968,878‐16,194,549	N/A	Maternal	Normal
18	Del	16p13.11	2,099,212	chr16:14,762,269‐16,861,481	N/A	Maternal	Normal
19	Del	16p13.11	1,400,814	chr16:14,910,227‐16,311,041	46,XX	De novo	N/A
20	Del	16p13.11	3,351,927	chr16:15,280,026‐18,631,953	46,XY	De novo	N/A
21	Del	16p13.11	1,225,723	chr16:14,968,855‐16,194,578	N/A	Maternal	Normal
22	Del	16p13.11	1,676,710	chr16:14,910,205‐16,586,915	N/A	De novo	N/A
23	Del	16p13.11	1,284,373	chr16:14,910,205‐16,194,578	N/A	De novo	N/A
24	Del	16p13.11	1,951,305	chr16:14,910,205‐16,861,510	46,XX	De novo	N/A
25	Del	16p13.11	1,595,848	chr16:14,715,193‐16,311,041	N/A	De novo	N/A
26	Del	16p13.11	1,342,163	chr16:14,968,878‐16,311,041	N/A	Maternal	Normal
27	Del	16p12.2	1,334,507	chr16:21,311,229‐22,645,736	N/A	De novo	N/A
28	Del	16p12.2	570,380	chr16:21,837,522‐22,407,902	N/A	Maternal	Normal
29	Dup	16p11.2	2,242,487	chr16:29,849,198‐32,091,685	N/A	De novo	N/A
30	Dup	16p11.2	516,614	chr16:29,673,954‐30,190,568	46,XY,inv(9)(p12q13)	De novo	N/A
31	Del	16p11.2	516,614	chr16:29,673,954‐30,190,568	46,XY	De novo	N/A
32	Del	16p11.2	516,614	chr16:29,673,954‐30,190,568	46,XY	De novo	N/A
33	Del	16p11.2	516,614	chr16:29,673,954‐30,190,568	46,XY,del(8)(p11.2)	De novo	N/A
34	Del	16p11.2	516,614	chr16:29,673,954‐30,190,568	N/A	De novo	N/A

Abbreviations: CNV: copy number variant; Del: deletion; Dup: duplication; N/A: not applicable.

### 3.1 Microdeletion/microduplication of 16p13.3

Four cases were diagnosed with 16p13.3 CNVs, including one with duplication and three with deletions.

The case with the duplication was a 1.03 Mb de novo mutation partially involved in the common 16p13.3 microduplication syndrome region (Marangi et al., 2008), without *CREBBP* involvement.

Two cases were diagnosed with 14–16 kb homozygous microdeletion of 16p13.3 involving *HBA1* and *HBA2*. Of these, one showed cardiomegaly and suspected dilated cardiomyopathy on prenatal ultrasound, whereas the other did not show any structural anomaly prenatally. The parents of both patients were confirmed as alpha‐thalassemia Southeast Asian deletion carriers. Both patients were diagnosed with thalassemia major and the mother underwent termination of pregnancy.

Another de novo microdeletion of 0.54 Mb in 16p13.3 is related to the gene encoding Tre‐2/Bub2/Cdc16 1 domain family member 24 (*TBC1D24*), a member of the Tre2‐Bub2‐Cdc16 (TBC) domain‐containing RAB‐specific GTPase‐activating proteins. The fetus carrying this mutation was terminated.

### 3.2 Microdeletion/microduplication of 16p13.11

**FIGURE 1:**
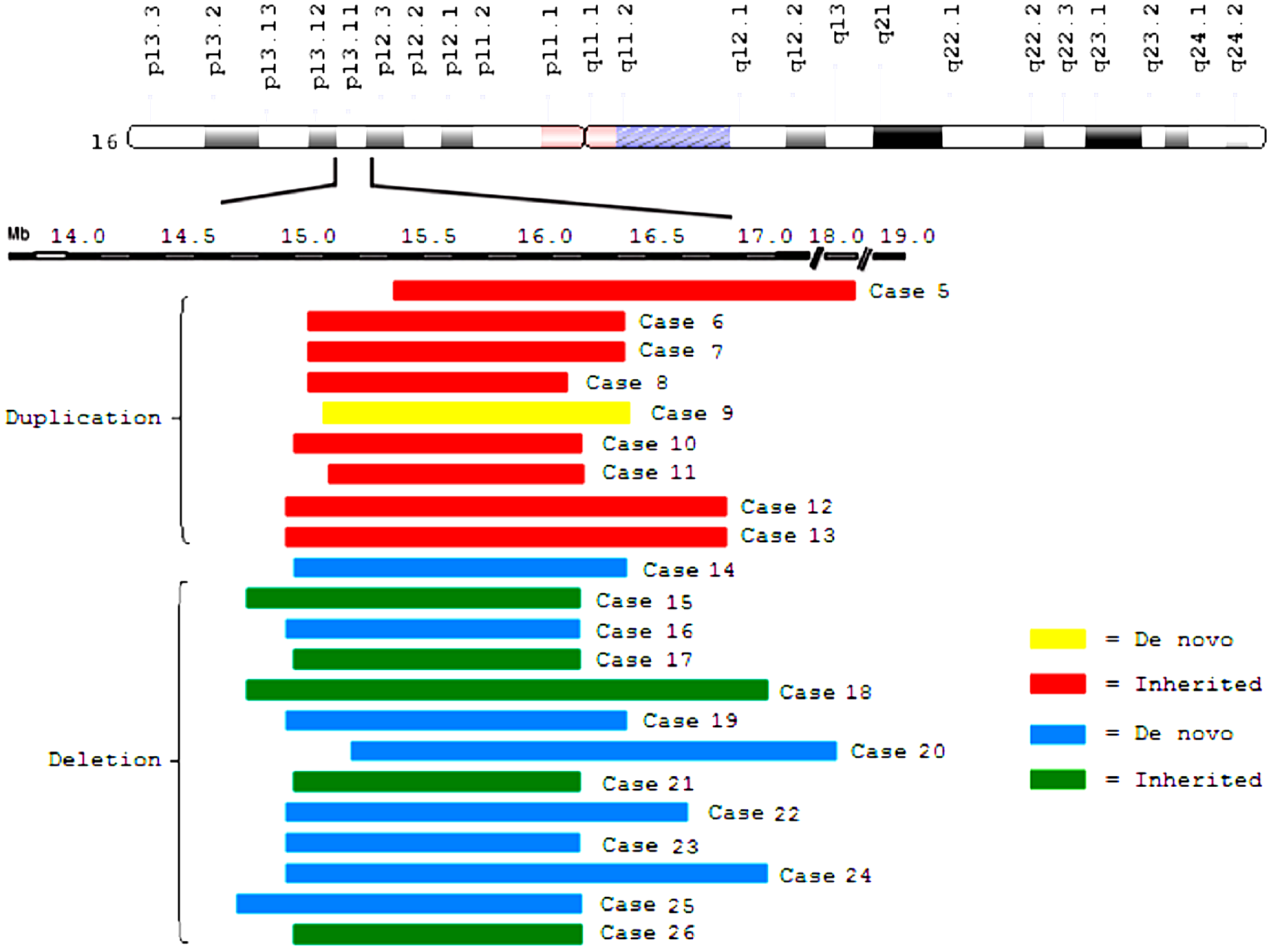
The extent and size of 16p13.11 copy number variantss. Only one case harbored a de novo duplication, while all others harbored duplications inherited from phenotypically normal parents. Eight of 13 deletion cases harbored de novo deletion, while five were inherited from phenotypically normal parents.

Of the 22 fetuses with 16p13.11 CNVs, 13 were diagnosed with microdeletions and nine with microduplications (Figure 1).

The size of the 16p13.11 microdeletion ranged from 1.23 to 3.35 Mb. The distal breakpoints of the deletions ranged from 14,715,193 to 15,280,026 kb, while the proximal breakpoints ranged from 16,194,549 to 18,631,953 kb (hg19/GRCh37). In five cases, mutations were inherited from a phenotypically normal mother and the other eight had de novo mutations.

The size of the 16p13.11 microduplication ranged from 1.05 to 3.27 Mb. The distal breakpoints of the duplications ranged from 14,910,228 to 15,148,986 kb, while the proximal breakpoints ranged from 16,127,848 to 18,669,696 kb (hg19/GRCh37). In eight cases, mutations were inherited from phenotypically normal parents—two maternal and six paternal. The remaining one had a de novo duplication.

Thirteen cases were delivered at term with 16p13.11 CNVs, including six with duplications and seven with deletions. Babies delivered with 16p13.11 microduplication ranging from 1.08 to 3.27 Mb were all inherited from a phenotypically normal parent. There were no major complications during newborn follow‐up, including neurodevelopmental disorders in any of the six cases with 16p13.11 microduplication. The other three patients with 16p13.11 microduplication were lost to follow‐up, including one diagnosed with cleft lip via prenatal ultrasound.

The size of the deletions in patients delivered with 16p13.11 microdeletion ranged from 1.23 to 3.35 Mb. Four microdeletions were de novo and three were inherited from the mother. One of the cases (case 17) was with a 1.23 Mb microdeletion inherited from a phenotypically normal mother who discovered a café‐au‐lait spot, while MRI reported her normal later. However, language delay was noted at the age of 3 years, with no DD currently noted. Another case (case 24) had a 1.95 Mb de novo microdeletion and developed a language delay at the age of three.

According to the parent's decision, termination of pregnancy was performed in four cases carrying 16p13.11 microdeletions and two cases were lost to follow‐up. All cases that received termination were de novo deletions, with sizes ranging from 1.28 to 1.68 Mb. No abnormal prenatal ultrasound findings were observed.

### 3.3 Microdeletion of 16p12.2

Two patients were diagnosed with a 16p12.2 microdeletion. One had a 1.33 Mb de novo deletion and the other had a 0.57 Mb maternally inherited microdeletion. The size of the deletion was between 0.57 and 1.33 Mb. The distal breakpoints of the deletions ranged from 21,311,229 to 21,837,522 kb, while the proximal breakpoints ranged from 22,407,902 to 22,645,736 kb. Both fetuses were delivered at term without obstetric complications and showed no neurodevelopmental disorders in the first 5 years of their lives.

### 3.4 Microdeletion/microduplication of 16p11.2

**FIGURE 2:**
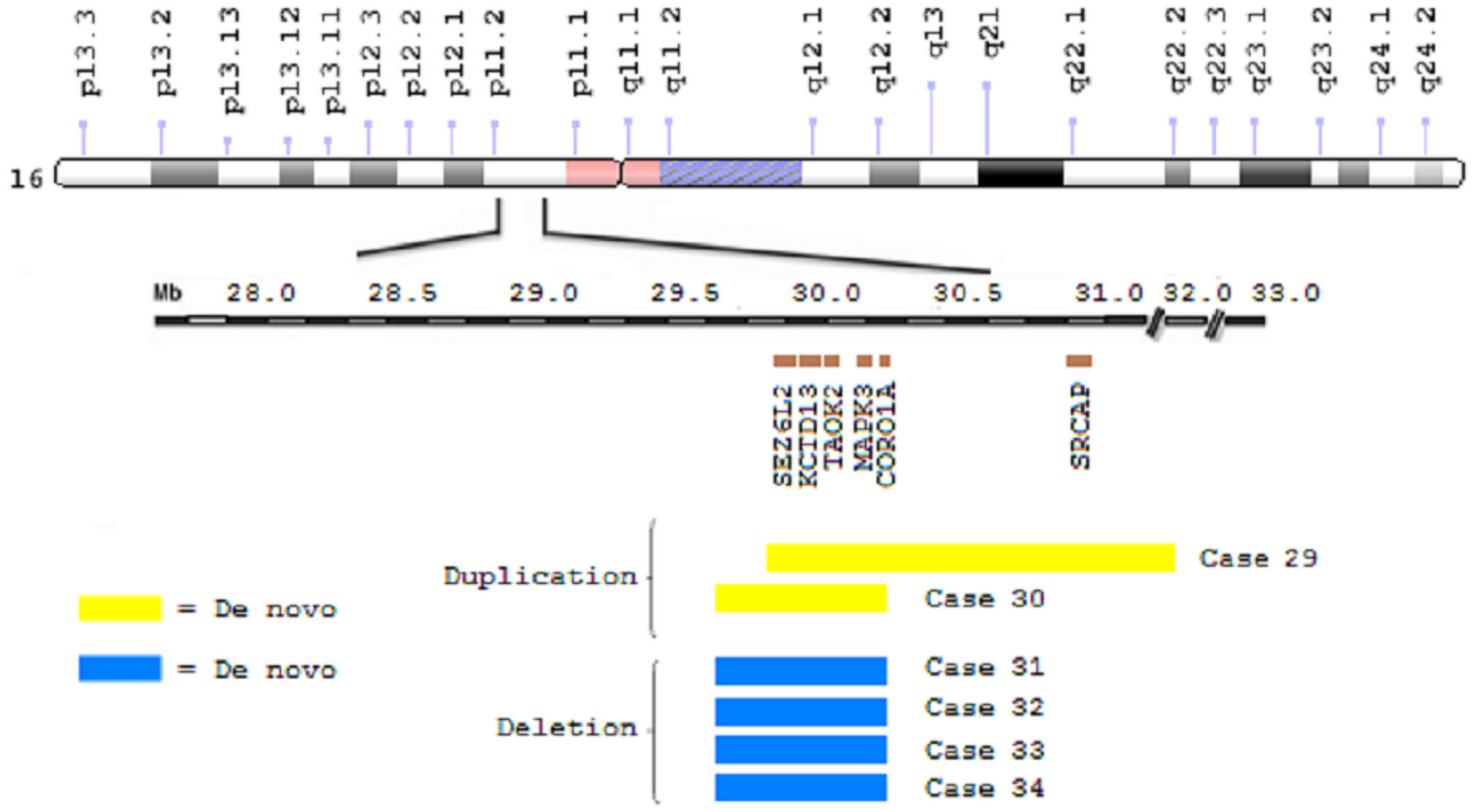
Size and genomic content of 16p11.2 deletions and duplications. Cases 29, 30, and 31 are duplications, and cases 32, 33, 34, and 35 are deletions.

Of the six fetuses referred, four were diagnosed with 16p11.2 microdeletions and two with 16p11.2 microduplications (Figure 2).

The size of the 16p11.2 microdeletion was approximately 0.52 Mb. The distal breakpoint of the deletions was at 29,673,954 kb, whereas the proximal breakpoint was at 30,190,568 kb (hg19/GRCh37).

All four 16p11.2 microdeletions were de novo and involved in 16p11.2 microdeletion syndrome. One fetus was terminated and one was lost to follow‐up. Two of the four cases delivered at term reported normal ultrasound findings prenatally, and the birth body weight was not small for gestational age. However, one patient (case 33) presented with poor fine and gross motor coordination at the age of two but no socioemotional development or language delay was noted. The case improved under early intervention. Another case reported no obvious neurodevelopmental disorders until the age of four. Although a clinical association between approximately 600 kb (593 kb) proximal deletions and duplications at 16p11.2 (breakpoint 4 and breakpoint 5 BP4‐BP5, between 29.5 and 30.1 Mb), and neurodevelopmental disorders has been reported (Torres et al., 2016), some deletions and duplications are still observed in normal individuals (Bijlsma et al., 2009).

The size of the 16p11.2 microduplication ranged from 0.52 to 2.24 Mb. The distal breakpoints ranged from 29,673,954 to 29,849,198 kb, while the proximal breakpoints ranged from 30,190,568 to 32,091,685 kb (hg19/GRCh37). All the two cases with duplication were de novo mutation.

The two cases with 16p11.2 microduplication were delivered at term. The one with a 2.24 Mb de novo duplication (case 29) was diagnosed with intrauterine fetal growth restriction by prenatal ultrasound and was born preterm at 34 weeks of gestational age, with a birth body weight of 1434 g (small for gestational age). No other obstetric or neurological complications were observed.

## 4 DISCUSSION

Chromosomal microarray analysis has been used for the first‐line analysis of neurodevelopmental disorders such as intellectual deficiency, autism, and DD in recent years (Manning et al., 2010). It is highly sensitive in detecting pathogenic CNVs and is beneficial for prenatal genetic counseling. However, there are still many unknown factors, such as gene penetrance and expressivity. Improvements in the array technique also make it difficult to explain cases of variants of unclear significance. Furthermore, CNVs discovered incidentally in healthy populations or inherited from apparently healthy parents, but with variant phenotypes, make prenatal counseling challenging.

Chromosome 16 is involved in many CNVs and multiple microdeletion/duplication syndromes. Some of these CNVs, such as 16p11.2 and 16p13.11, are pathogenic while the pathogenicity of the others is still debatable as they have variable phenotypes. Our analysis involved various diagnosis techniques, including prenatal examinations and postnatal follow‐up, of fetuses with chromosome 16p CNVs.

Duplication involving the RTS region is called 16p13.3 microduplication syndrome (Demeer et al., 2013). We report a case harboring a duplication involving common 16p13.3 duplication region, without *CREBBP*. The region is associated with familial Mediterranean fever and the Beaulieu‐Boycott‐Innes syndrome caused by *THOC6* mutation, which causes DD, ID, and dysmorphic facial features. Other developmental anomalies, such as cardiac and renal defects, cleft palate, and corpus callosum dysgenesis may also be present (Beaulieu et al., 2013; Boycott et al., 2010). However, no similar duplication cases have been reported prenatally. The fetus was terminated according to the parent's decision.

CNVs involving 16p13.11 are also found in phenotypically normal populations. The pathogenicity of duplication is still uncertain, while that of 16p13.11 microdeletion is more established. For patients with deletions involving 16p13.11, microcephaly and DD have been previously reported, while duplication has been reported to be consistent with behavioral abnormalities, cognitive impairment, and congenital cardiac defects (Nagamani et al., 2011). Despite phenotypic variability, de novo microdeletions are likely to cause pathological phenotypes in offspring, whereas the role of duplications in clinical phenotypes is uncertain and may be benign (Hannes et al., 2009). However, increasing evidence has shown a relationship between 16p13.11 duplication and cognitive impairment, suggesting that it may be pathogenic (Allach El Khattabi et al., 2020). In our cohort, eight of nine fetuses with 16p13.11 duplication were inherited from their parents, and six inherited cases delivered at term without complications and showed no postnatally observed DD. A previous study reported 16p13.11 microduplications are likely pathogenic when detected in the context of DD/ID/ASD even with phenotypically normal parent. In the cohort, 89% of cases were inherited and at least 50% of cases have parents with normal phenotype (Allach El Khattabi et al., 2020). However, our study reported a high percentage of inherited cases with normal postnatal development, which might be a new perspective in prenatal counseling.

The two fetuses that harbored 16p12.2 deletion showed no structural abnormalities on prenatal ultrasound. However, 16p12.2 deletion cases have been reported to be consistent with congenital heart disease and cleft lip (Khau Van Kien et al., 2005). The one with small 16p12.2 deletion (570 kb) involved one OMIM gene (*UQCRC2*). The two cases also carried de novo deletions in another autosomal recessive deafness gene (*OTOA*). However, both cases did not show any symptoms of neurodevelopmental disorders postnatally. A small number of cases leads to difficulty in interpreting incomplete penetrance and expressivity.

16p11.2 is known to be involved in neurodevelopmental disorders. Deletion and duplication have distinct characteristics, including mirroring phenotypes such as head size and body mass index (Steinman et al., 2016). 16p11.2 microdeletion manifests a wide variety of phenotypes, including neurodevelopmental disorders such as ASD, ID, behavioral disorders, seizures/epilepsy, and hypotonia, as well as congenital malformations of the spine, brain, and cardiovascular system, such as macrocephaly and Chiari I/cerebellar tonsillar ectopia (Bernier et al., 2017; Jacquemont et al., 2011; LeBlanc & Nelson, 2016; Steinman et al., 2016). Another case report showed a rare association with congenital diaphragmatic hernia (Genesio et al., 2018). Some of the symptoms discovered in microdeletion syndrome are also seen in duplication cases and include speech articulation abnormalities, hypotonia, abnormal agility, and seizures/epilepsy (Steinman et al., 2016). Autism, schizophrenia, anorexia, microcephaly (D'Angelo et al., 2016; Horev et al., 2011), cerebral white matter/corpus callosum abnormalities, and ventricular enlargement (Steinman et al., 2016). However, individuals with duplications have higher frequencies of cognitive impairment, such as intellectual impairment, similar frequencies of ASD, and lower chances of psychiatric disorders (D'Angelo et al., 2016).

Macrosomia is a common postnatal characteristic in patients with 16p11.2 microdeletions, but it is doubtful that this characteristic would be prominent in prenatal ultrasound and be predictive for further genetic testing (Steinman et al., 2016). In our study, two cases delivered with 16p11.2 microdeletions reported normal biparietal diameter and head circumference prenatally, and no obvious macrosomia was noted prenatally. Thus, determining if macrosomia could be a prognostic factor requires the analysis of more cases. Macrosomia may be prevalent in microdeletion cases, while macrosomia is common in microduplication cases, probably due to differential *KCTD13* expression (Golzio et al., 2012).

To the best of our knowledge, no other large stud has undertaken prenatal diagnosis and postnatal follow‐up of patients with 16p11.2 mutations. Skeletal malformation is one of the most frequent prenatal findings discovered recently (Lin et al., 2018), but more cases need to be evaluated owing to its rarity. Deletion is associated with congenital malformations including skeletal, cardiovascular, urogenital, and central nervous system malformations. However, owing to the small number of cases, no common congenital malformations were noted in our study.

One de novo case (case 29) of 2.24 Mb microduplication was discovered prenatally with fetal growth restriction, while postnatal findings were only small size for gestational age, and no other neurodevelopmental disorders developed afterward. Another case (case 30) with a de novo duplication involving the common 16p11.2 region was also born without major complications, and no other DD was reported. Thus, the size of the duplication is not the only factor governing prognosis.

Of the 34 cases of CNVs in chromosome 16, only four had abnormal sonographic findings prenatally, including cleft lip, echogenic intracardiac focus, intrauterine growth restriction, and cardiomyopathy‐related cardiomegaly. Previous studies have shown that certain CNV regions, including 22q11.2, 7q11.2, 8p23, 1q21.1, 16p13.11, 15q11.2, and 2p13.3, are associated with congenital heart disease. Common cardiovascular malformations, such as coarctation of the aorta, ventricular septal defects, double outlet right ventricle, pulmonic stenosis, and hypertrophic cardiomyopathy (Glessner et al., 2014; Greenway et al., 2009; Zaidi & Brueckner, 2017). However, in our 16p13.11 population, only cleft lip and echogenic intracardiac foci were detected. Microcephaly and macrocephaly are related to 16p11.2 CNVs (Steinman et al., 2016), however, we found only one case carrying a 16p11.2 microduplication with intrauterine growth restriction. Thus, sonographic features do not seem to be significant in prenatal diagnosis and prognostic prediction. Table 3 provided a detailed genotype–phenotype correlation of all CNVs reported in our cohort, and the results of previous studies.

**TABLE 3:** The genotype–phenotype correlation in prenatal and postnatal period of chromosome 16 short arm CNVs.

		Prenatal ultrasound finding		Postnatal finding	
		Our patients	Previous reports	Our patients	Previous reports
16p13.3	Dup	—	Dysmorphic facial features, cleft palate^a^ ^,^ ^b^	—	Dysmorphic facial features, cardiac and renal defects, cleft palate, and corpus callosum dysgenesis^a^ ^,^ ^b^
	Del	Cardiomegaly	N/A	—	Hypertelorism, broad or prominent nasal bridge, and talipes equinovarus^c^
16p13.11	Dup	Cleft lip	Congenital heart disease^d^	—	Cardiac defects, skeletal malformation^d^
	Del	Echogenic intracardiac focus	Microcephaly, agenesis of the corpus callosum, fetal brain disruption^e^	Café‐au‐lait spot	Microcephaly^d^
16p12.2	Del	Oligohydramnios	Congenital heart disease, cleft lip^f^ ^,^ ^g^	—	Cardiac defects, renal abnormalities, and cleft lip^f^
16p11.2	Dup	Intrauterine growth restriction	N/A	Small of gestational age	Microcephaly^h^ ^,^ ^i^
	Del	—	Skeletal malformation, congenital diaphragmatic hernia^j^	—	Macrosomia, Skeletal, cardiovascular, urogenital, and central nervous system malformations^h^ ^,^ ^i^

Abbreviations: CNV: copy number variant; Del: deletion; Dup: duplication; N/A: not applicable.

^a^ Beaulieu et al. (2013).

^b^ Boycott et al. (2010).

^c^ Breuning et al. (1993).

^d^ Nagamani et al. (2011).

^e^ Paciorkowski et al. (2013).

^f^ Khau Van Kien et al. (2005).

^g^ Linnane et al. (2021).

^h^ Bernier et al. (2017).

^i^ Steinman et al. (2016).

^j^ Lin et al. (2018).

Our study is limited in that it investigated a small population. Moreover, the follow‐up period was too short and may have led to undetected disorders with intellectual or learning disabilities and behavioral issues, such as autism. Thus, the follow‐up period should be extended so that growth and development can be evaluated more thoroughly in the future.

Increasing evidence supports the pathogenicity of the 16p13.3 and 16p11.2 CNVs. Incomplete penetrance and variable expressivity make prenatal counseling challenging for other chromosome 16 CNVs. Most cases with inherited 16p13.11 microduplication were reported to have normal development in early childhood, and we also report a few cases of de novo 16p CNVs without further neurodevelopmental disorders.

## AUTHOR CONTRIBUTIONS

**Jessica Kang** contributed to drafting the manuscript. **Chih‐Ling Chen** and **Yi‐Yun Tai** organized and analyzed patient data. **Han‐Ying Chen** prepared and formatted the tables and figures. **Chien‐Nan Lee** and **Yi‐Ning Su** helped in design the study and in data collection. **Shin‐Yu Lin** conceptualized the study and revised the manuscript. All authors have read and approved the final manuscript.

## FUNDING INFORMATION

This work was supported by the Ministry of Science and Technology under Grant 108‐2314‐B‐002‐143‐MY3 and the National Taiwan University Hospital under Grant NTUH108‐N4040.

## CONFLICT OF INTEREST STATEMENT

There is no conflict of interest.

## ETHICS STATEMENT

All the research methods used in this study were approved by the National Taiwan University Hospital Research Ethics Committee (201801010RINC).

## Data Availability

Research data are not shared.
